# Sodium Reduction in Restaurant Food: A Randomized Controlled Trial in China

**DOI:** 10.3390/nu14245313

**Published:** 2022-12-14

**Authors:** Wenwen Du, Puhong Zhang, Jiguo Zhang, Yuan Li, Feng J. He, Xiaofan Zhang, Chang Su, Xiaofang Jia, Li Li, Jing Song, Bing Zhang, Huijun Wang

**Affiliations:** 1 National Institute for Nutrition and Health, Chinese Centre for Disease Control and Prevention, Beijing 100050, China; 2 Key Laboratory of Trace Element Nutrition, National Health Commission, Beijing 100050, China; 3 George Institute for Global Health, Peking University Health Science Center, Beijing 100600, China; 4 Faculty of Medicine, University of New South Wales, Sydney, NSW 1466, Australia; 5 Wolfson Institute of Population Health, Barts and The London School of Medicine and Dentistry, Queen Mary University of London, London E1 4NS, UK

**Keywords:** sodium, restaurant food, randomized controlled trial, China

## Abstract

Restaurant food is one of the important sources of sodium intake in China. We aimed to determine whether a restaurant-based comprehensive intervention program may induce lower sodium content in restaurant food. A randomized controlled trial was implemented between 2019 and 2020 in 192 restaurants in China. After baseline assessment, the restaurants were randomly assigned to either an intervention or a control group (1:1). Comprehensive activities designed for intervention restaurants were conducted for one year. The primary outcome was the difference in change of sodium content estimated by the mean values of five best-selling dishes for each restaurant, from baseline to the end of the trial between groups. In total, 66 control restaurants and 80 intervention restaurants completed the follow-up assessment. The average sodium content of dishes at baseline was 540.9 ± 176.8 mg/100 g in control and 551.9 ± 149.0 mg/100 g in intervention restaurants. The mean effect of intervention after adjusting for confounding factors was −43.63 mg/100 g (95% CI: from −92.94 to 5.66, *p* = 0.08), representing an 8% reduction in sodium content. The restaurant-based intervention led to a modest but not significant reduction in the sodium content of restaurant food. There is great urgency for implementing effective and sustainable salt reduction programs, due to the rapid increase in the consumption of restaurant food in China.

## 1. Introduction

Strong evidence has shown that high dietary sodium intake is associated with elevated blood pressure (BP) and an increased risk of cardiovascular diseases (CVDs), and that a reduction in sodium intake could lower BP and reduce BP-related diseases [1,2,3,4]. According to the health effects of dietary risks in 195 countries, more than half of diet-related deaths and two-thirds of diet-related disability-adjusted life years (DALYS) were attributable to high intake of sodium, and low intake of whole grains and fruits. High intake of sodium was the leading dietary risk for deaths and DALYs in China [5]. A large sample of China using 24-h urinary data showed the average sodium was 4318.1 mg/d (equivalent to 11.0 g/d of salt) in 2018 [6], which greatly exceeds the recommended limit of 5 g/d set by the Chinese Dietary Guidelines [7] and the World Health Organization (WHO) [8]. China’s burden of mortality, morbidity, and disability from hypertension is the highest in absolute and relative terms globally. The last (2012–2015) national hypertension survey showed that the overall prevalence of hypertension and prehypertension was 23.2% and 41.3%, respectively [9]. The major source of sodium in the Chinese diet is the salt and sauces added during cooking at home and at the restaurant, which accounted for almost 80% of the total sodium intake [10,11]. Changes in the food environment caused by urbanization and globalization have led to a rise in eating away from home [12]. The sodium content of dishes in restaurants was generally high [13,14,15] and higher than dishes prepared at home [16]. A survey of restaurants in six provinces of China showed that 49.9% of dishes contained more than 2400 mg of sodium per serving [15], and chefs preferred to use more kinds of salty condiments to achieve flavors. Those who ate out tend to have a higher dietary sodium intake than people who ate at home [17], and the more often people ate out, the higher their sodium intake [18]. Therefore, reducing the sodium content of restaurant dishes is an important step in reducing the population’s sodium intake and further reducing the burden of disease and death associated with a high-sodium diet.

Sodium reduction is one of the most cost-effective strategies for chronic disease prevention and control recommended by WHO. A cost-benefit analysis of government sodium reduction strategies in 183 countries found that sodium reduction interventions are highly cost-effective, with an estimated 10 percent reduction in sodium intake over 10 years in each country averting approximately 5.8 million disability-adjusted life years related to cardiovascular disease per year at a population-weighted average cost of only I$1.13 [19].

As the restaurant industry contributes more and more to people’s sodium intake, many countries have started to develop strategies to reduce sodium in restaurants. A systematic review on international sodium reduction policies in restaurants showed that menu labeling, target setting, and reformulation were the most popular strategies. However, the evaluations of these policies were limited and showed inconsistent results [20]. Menu labeling and target-setting are usually implemented in chain restaurants in Western countries, but seeming to be difficult to work for most restaurants in China. Moreover, in most Chinese restaurants, the chefs or cooks determine the amount of salt and salt-rich sauces to be added to dishes, and the amount of salt or sauces is based on the cook’s experience rather than weighing. Therefore, reformulation findings in other countries may provide more insights. Studies using flavored salt confirmed the important role of Mediterranean herbs and spices in enhancing saltiness perception [21,22]. The feasibility and effectiveness of the potential strategies need to be further explored. 

To tackle high sodium content in restaurant foods, we have developed a comprehensive intervention package following the social cognitive theory. To test whether the intervention package could achieve a sustainable sodium reduction in Chinese restaurant foods, we carried out a randomized controlled trial.

## 2. Materials and Methods

### 2.1. Study Design

A randomized controlled trial, with restaurants as study participants, was carried out between January 2019 and December 2020. Comprehensive intervention activities were conducted only in restaurants of the intervention group. Baseline and follow-up assessments were launched at the beginning and end of first-year comprehensive intervention based on the average sodium content of 5 best-dishes of the restaurant by laboratory test. In addition to the laboratory test, the basic information and salt reduction services among the restaurants, salt-related knowledge, attitude, and practice among restaurant consumers were collected by trained local investigators through a mobile electronic data capture system (EDC), at baseline, mid-term, and end-term points. The local CDC investigators responsible for data collection and monthly supervision records. Details of the method have been published elsewhere [23].

### 2.2. Participants

The participant’s restaurants came from 12 counties of 6 provinces in China: Qinghai province (Chengbei and Chengzhong), Heilongjiang province (Longfeng and Saertu), Hebei province (Zhengding and Zhaoxian), Hunan province (Yuhua and Kaifu), Sichuan Province (Wuhou and Guangan), and Jiangxi province (Xinjian and Xihu). The counties in each province had similar socioeconomic levels. From each county, we recruited 16 restaurants specializing in Chinese cuisine, including 4 large-size restaurants, 8 medium-size restaurants, and 4 small-size restaurants. We code the restaurant size according to the business scale [23]. 

Inclusion criteria were as follows:

Restaurants specializing in Chinese cuisine.
Restaurants that agreed to participate in 1-year intervention and at least two assessments.
Restaurants with complete records of salt and other condiments purchased and used.
Restaurants that had been operating at least 1 year and without plans to move out or close in the next 2 years.
More than 50% of dishes in the restaurant are cooked in the restaurants rather than centrally prepared and provided.
Restaurants that had been involved in other sodium reduction programs were excluded.

### 2.3. Randomization and Masking

This trial was open-label. Restaurants staff, consumers, and local investigators in recruitment and baseline data collection were blinded to the group allocation until the commencement of the intervention. After baseline assessment, a computer-generated randomization system allocated restaurants to either intervention or control group (1:1) in each county, stratified by the size of restaurants. 

### 2.4. Procedures

The framework of the comprehensive intervention was designed following the social cognitive theory, which considered that behaviors were influenced by the interaction of personal and environmental factors. Detailed descriptions of the intervention activities were provided in Supplement Table S1. Mainly targeting four aspects to reduce sodium content in restaurant foods as follows:

(1) Building supportive environment: 

Materials on sodium reduction such as videos, posters, brochures, leaflets, and table displays were delivered regularly to restaurants and displayed at noticeable positions, including information on sodium and health, as well as available reduced-sodium services. The supportive environments built during the whole trial period made it easier for customers to choose lower sodium options. 

(2) Activities on consumers:

In each intervention restaurant, the activities on consumers included three parts. First, Materials in supportive environment convey the idea “less salt more health” to the consumers. Second, promoted consumers to request salt reduction services by providing multiple choice for salt used (normal, 70% slat, or 50% salt). Third, marked lower-sodium dishes on the menu so as to guide consumers to make lower-sodium choices. The marked menu was used throughout the 12-month trial period. 

(3) Enhancing chefs’ cooking skills to reduce sodium:

We invited culinary experts to develop a series of training materials for chefs, comprising lessons on the following: ‘sources of sodium in restaurant meals’, ‘why reducing sodium’, and ‘practical cooking skills to reduce sodium’. A face-to-face training by a professional team for the target chefs of each county was organized at least once a year. Local investigators were responsible for enhancing the knowledge and skills in each intervention restaurant through monthly follow-up supervision. The intervention restaurants were encouraged to reduce sodium use by at least 10% in all dishes and more reductions according to customers’ request. 

(4) Encouraging waiters/waitresses to proactively remind and introduce lower-sodium choices in ordering services:

We invited culinary experts to develop a series of training materials for service staff of restaurants, comprising lessons on the following: ‘sources of sodium in restaurant meals’, ‘environment building’, and ‘service and communication skills’. A face-to-face training by a professional team for the target waiters/waitresses of each county was organized at least once a year. Local investigators were responsible for enhancing the knowledge and skills in each intervention restaurant by monthly follow-up supervisions. Service staff were trained to recommend customers the lower-sodium dishes, as well as remind them the reduced-sodium option for other orderings in their daily work.

Due to the COVID-19 pandemic in the early 2020, restaurants were closed for several months. We evaluated the influences of pandemic on intervention activities, including interruption time, staff turnover, willingness, and challenges to continue the program. After an average interruption of 4 months, the intervention period was extended accordingly, all the intervention activities continued, so as to ensure that the total intervention duration was 12 months. 

Restaurants in the control group did not receive any intervention.

### 2.5. Outcomes

The primary outcome was the difference between the intervention and control groups in the sodium content change of restaurant dishes from baseline to the end of trial. The sodium content of dishes in a restaurant was estimated by the mean sodium content of five best-selling dishes for a restaurant. The secondary outcome was the difference between intervention and control groups in the change of the amount of salt and main salty condiments used by chefs, and the proportion of less-sodium dishes ordered by the customers. All outcomes were assessed at baseline and the end of trial with an identical method for all the restaurants. For the primary outcome, the whole portions of five best-selling dishes including sauce and soup when appropriate in each restaurant were anonymously collected at baseline and end of trial. The sodium content was tested by laboratory flame atomic absorption spectrometry method in the same lab, without identifying information of group and trial period. Theoretically, we collected the same dishes at baseline and follow-up assessments. However, if the dishes collected at baseline were not available at the follow-up assessment, comparable dishes with similar ingredients and cooking methods were chosen instead.

For the secondary outcomes, the amounts of food ingredients as well as salt and other condiments used in all dishes (up to 50 dishes with top sales volume per restaurant) were recorded, assisted by electronic food scale. The use of salt and other condiments (g/100) in all dishes was then calculated. “What is the proportion of orders requiring less salt when customers order in the restaurants?” were interviewed by owners/managers who directly manage the restaurant and asked to choose one option from “0, 1–10%, 11–20%, 21–30% or 31%−”. 

For the feasibility outcomes, at the middle period of intervention, i.e., after the 4-months interruption of intervention due to COVID-19, implementation of nine key intervention activities was anonymously assessed by local investigators. 

### 2.6. Statistical Analysis

Based on the results of a previous study in Chinese restaurants [24], and assuming an SD of 1000 mg/100 g for the sodium content in Chinese restaurant dishes, we estimated that a sample size of 192 restaurants would achieve 80% power (α = 0.05) to detect a difference in average sodium content for the five best-selling dishes by 196.85 mg/100 g dish between the two groups, allowing for a 20% dropping rate of restaurants. Therefore, a total of 192 restaurants were recruited for outcome assessments.

Data analysis was performed according to the intention-to-treat approach, that is, restaurants that completed the baseline assessment were analyzed according to their randomly assigned group. We tested the effects of the intervention on outcomes by using mixed linear models. The models included random intercept and fixed effect variables (e.g., time, group, time × group interaction, and covariates). The time × group interaction term indicated the differential change by group from baseline to the follow-up assessment. We Adjusted for restaurant size, area, cost, and turnover. We also carried out a subgroup analysis by restaurant size and location area.

Two sensitivity analyses were performed to examine the robustness of the intervention effect on the primary outcome. The first analysis included only restaurants that completed both baseline and follow-up assessments. The second analysis included all restaurants at baseline using Multiple Imputation (MI).

Results were reported as means, standard deviations, and 95% confidence intervals where appropriate. All analyses were two-sided and *p* < 0.05 was considered significant. The statistical analyses were performed using SAS software, version 9.4 (SAS Institute).

### 2.7. Patient and Public Involvement

Restaurants’ managers, chefs, waiters/waitresses, and consumers were involved in the design and conduct of the study. At the protocol development and pilot stage, group interviews were held to gain their opinion on the feasibility and willingness of the comprehensive salt reduction intervention in restaurant settings. Furthermore, culinary experts helped us to develop a series of training materials, including training courses, manuals and videos, as well as to offer face-to-face training for staff of interventional restaurants in each county. Once the study is published, related restaurant managers, chefs, waiters/waitresses, and consumers will be informed of the findings by the local CDCs who responsible for the organization and implementation of the restaurant intervention. 

### 2.8. Ethics Committee Approval 

The study protocol was approved by Queen Mary (University of London) Ethics of Research committee (QMERC2018/14) and the Review Board of the National Institute for Nutrition and Health, China CDC (20180314). The study was registered at chictr.org.cn (Chinese Clinical Trial Registry ChiCTR1800019694).

## 3. Results

**Figure 1:**
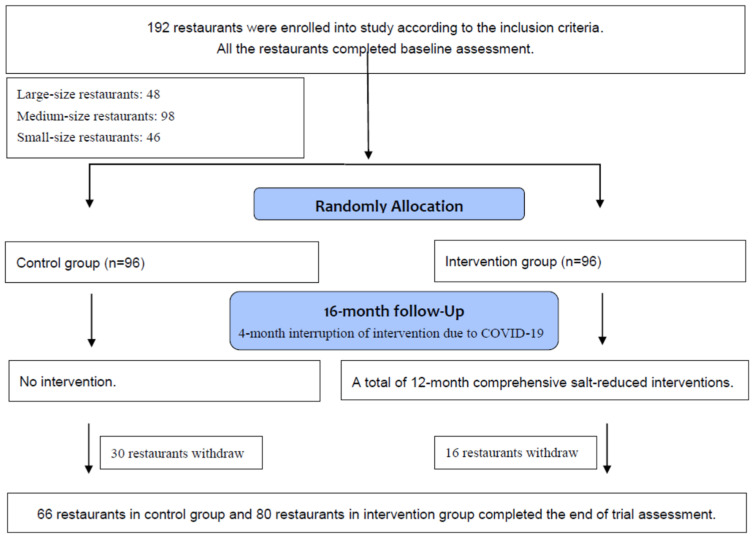
Trial profile of restaurant-based intervention to reduce salt in China.

We recruited 192 restaurants into this study (2 counties in each of six provinces, 16 restaurants from each county) according to the inclusion criteria into this study. During the trial, 30 (31.25%) restaurants in the control group and 16 (16.67%) restaurants in the intervention group were lost to follow-up, owing to closed down or unable to attend the end-of-trial assessment (Figure 1).

### 3.1. Characteristics of the Study Restaurants

Baseline characteristics of the study restaurants were similar between the two randomized groups on location area, restaurant size, average cost, monthly turnover, restaurant environment, and service (Table 1). One-third of the restaurants were located in the north, central, and south area, respectively. About half of the restaurants were medium-sized. In total, 37.50% of restaurants reported the availability of nutritional materials, and only 16.15% of restaurants provided less salt choice on the menu at baseline. The characteristics between the follow-up restaurants and lost-to-follow restaurants also showed no significant differences (Supplement Table S2).

### 3.2. Effects of Intervention on Sodium Content

Table 2 shows the results for sodium content by laboratory test. Based on the intention-to-treat analysis, the mean baseline sodium content was 540.9 (standard deviation 176.8) mg/100 g in the control group and 551.9 (149.0) mg/100 g in the intervention group. After the 12-month study, sodium content decreased in both groups, but to a greater extent in the intervention group (Table 2). The adjusted mean effect comparing the intervention group with the control group was −43.63 mg/100 g (95% confidence interval from −92.94 to 5.66, *p* = 0.08), which represents an 8% reduction in the sodium content. The results also report two sensitivity analyses in Table 2. The mean effect on sodium content was essentially unchanged, either the analysis included only 146 restaurants that completed sodium content tests of dishes both at baseline and at the end of the trial or whether the analysis included all 192 restaurants at baseline using MI.

Table 3 shows the subgroup results for sodium content by laboratory test. The adjusted mean effect on sodium content was −69.18 mg/100 g (from −164.74 to 26.37) in large-size restaurants, −40.45 mg/100 g (from −110.00 to 29.08) in medium-size restaurants, and −17.98 mg/100 g (from −128.32 to 92.35) in small-size restaurants, respectively. No significant difference was found between any of the subgroups in the intervention effect on sodium content. 

### 3.3. Effects of Intervention on Use of Salt and Salty Condiments

Table 4 shows the use of salt and salty condiments in restaurant dishes, based on the intention to treat analysis. During the study, the use of all types of salt and salty condiments decreased in the intervention group. In the control group, the only the use of monosodium glutamate and other condiments decreased. The adjusted mean effects on the use of salt, monosodium glutamate, soy sauce, and other condiments were −0.09 g/100 g (from −0.25 to 0.07), −0.06 (from −0.16 to 0.05), −0.08 (from −0.21 to 0.05), and −0.22 (from −0.69 to 0.25), respectively. 

### 3.4. Effects of Intervention on Less-Sodium Ordering

**Figure 2:**
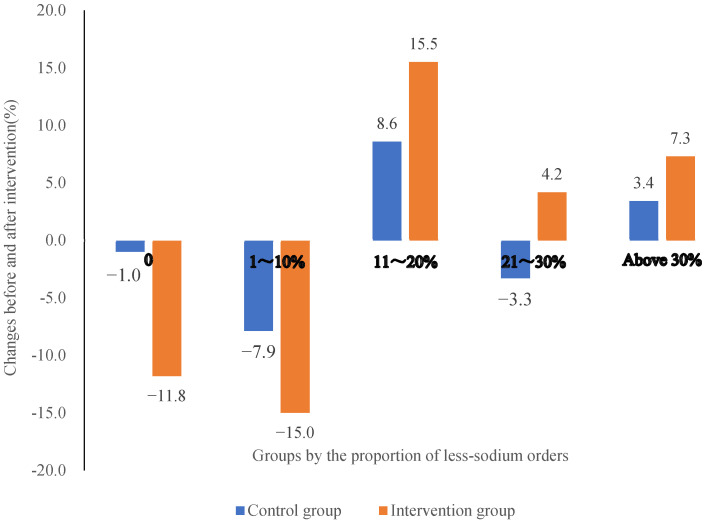
Comparison of less -sodium orders between control and intervention groups of restaurants before and after the intervention.

In the intervention group, the proportion of restaurants with above 10% of orders requiring less sodium increased by 27.0 percentage points after the intervention, while that in the control group had a cumulative increase of 8.70 percentage points (Figure 2). After the intervention, restaurants in the intervention group were 2.53 times more likely to increase less-sodium orders than those in the control group (odds ratio: 2.53, 95% CI: 1.09–5.90, *p* < 0.05) even after adjusting restaurant size, area, cost, and turnover.

### 3.5. Implementation of the Intervention Activities

At the midway point through the intervention, i.e., after the 4-month interruption of the intervention due to COVID-19, 78 restaurants in the intervention group were selected to investigate the implementation rate of each intervention activity (Supplement Figure S1). It was found that 91% of chefs reduced cooking salt when consumers requested less sodium, which was the intervention with the highest implementation rate. However, only about half of the restaurant staff actively introduced dishes with lower-sodium labels, which was the intervention with the lowest implementation rate. Moreover, the proportion of waiters asking consumers whether they would like the reduced sodium dishes is also low (at 64.1%). About 34.6% of the restaurants in the intervention group performed all nine interventions, and more than half of the restaurants performed seven or more interventions (Supplement Figure S1).

## 4. Discussion

### 4.1. Main Findings

In the 12-month study, we found that the sodium content of restaurant dishes decreased in both groups, but to a greater extent in the intervention group. There was no statistical significance in the adjusted mean effect comparing the intervention group with the control group. The lack of a significant effect could be due to various factors. Firstly, China has created a supportive social environment for salt reduction. Healthy China 2030 and the National Nutrition Plan (2017–2030) all have set the goals for salt reduction, which may result in a positive influence on all restaurants. Secondly, the proportion of fewer sodium requirements when eating out in restaurants increased during the COVID-19 epidemic [25], which means consumers’ health awareness has increased and accordingly chefs reduced adding salt in the dishes.

In this study, it was found that the proportion of less-sodium orders increased in both the intervention and non-intervention restaurants, but restaurants in the intervention group were 2.53 times more likely to increase their less-sodium orders than the control group. This may be related to the increasing willingness and behavior of consumers to reduce sodium [25]. In addition, the comprehensive intervention package involved in this study includes intervention measures targeted at consumers. For example, placing lower-sodium dishes on the menus and guiding consumers to make lower-sodium choices may also increase the proportion of less-sodium orders. However, at the dish level, there was no statistical difference in sodium content reduction between the intervention group and the control group. One of the possible reasons is that there are barriers to information transmission in the complex chain from consumers to waiters and then to chefs, so consumers’ demand for less sodium is not reflected in the effective reduction of sodium content in dishes. For example, the chefs are used to determining whether a dish is salty according to their own taste. It is difficult to reduce salt according to the requirements of consumers, because they think that it may not be delicious. In addition, it may also be related to the low overall proportion of less-sodium orders, which has not greatly reduced the sodium content of dishes. After the intervention, for example, only 12.5% of restaurants in the intervention group had above 30% less-sodium orders.

### 4.2. Comparison with Other Studies

The change in sodium content in restaurant foods can reflect the effect of intervention measures. Many countries have taken the sodium content of restaurant foods as the monitoring index to evaluate salt reduction intervention measures and set the decrease in sodium content in foods as the target of salt reduction intervention [26]. However, it is difficult to compare our results with other studies because of the different targeted foods and restaurant meal categories [27]. On the other hand, saltiness perception is also a combination of taste, smell, and the trigeminal response. Studies using flavored salt with Mediterranean aromatic plants instead of normal salt confirmed the role of herbs and spices in potentiating saltiness perception [21,22]. Therefore, evaluations based on different indicators could bring more uncertainty in comparisons of other research.

There was little evidence of the effects of salt reduction in restaurants. Findings from restaurant-based interventions for promoting healthy behaviors were not consistent. A cluster randomized trial implemented eight-week interventions in eight sit-down restaurants offering American or Latino dishes to explore the effects of healthy child menu items on meal purchasing behaviors for/by children [28]. The results found that sales of healthy child menu items were higher in restaurants receiving strategically designed interventions (menu plus, including training and promotional materials) versus restaurants receiving only menu interventions (menu-only), suggesting that the multi-component interventions may promote healthier child menu item behaviors. A pilot randomized community trial was conducted to test whether a community-level intervention in restaurants (7 in the intervention group vs. 7 in the control group) could improve the nutrition environment and promote healthy eating in restaurants located in two U.S. rural communities [29]. The results indicated that 10-month multiple interventions, including increasing availability, point-of-purchase/labeling, and promotion of healthier items, significantly increased the nutrition environment score; however, had no or limited effects on customer behaviors. The Healthy Chinese Take-Out Initiative, a sodium-reduction intervention among 206 Chinese take-out restaurants in low-income neighborhoods in Pennsylvania, was designed to reduce sodium in prepared dishes through training chefs [30]. The sodium content of the 3 most frequently ordered dishes was analyzed from baseline to 36 months after baseline among 40 restaurants. Significant reductions in the sodium content of all 3 dishes 36 months after a less-sodium cooking training intervention was observed. However, the chefs’ and owners’ perceptions of the need for sodium reduction and their self-efficacy for lowering sodium use returned to baseline levels 36 months later.

The inconsistent findings may be caused by different study designs, such as sample size, assessment outcomes, intervention activities, and the duration, as well as density. Comprehensive interventions based on system design were believed to be more effective at promoting healthy behaviors. However, the sustainable effects may vary. Our results indicated that 12-month comprehensive interventions in restaurants have little effect on the sodium reduction of dishes and the use of salt and salty condiments but play significant roles in promoting consumers’ behaviors to order less sodium. Due to the global epidemic of COVID-19, 4-month interventions occurred in the period, and nearly 24% of restaurants lost follow-up, which might decrease the power of testing a significant effect.

The restaurant is an important but complicated context for implementing healthy eating promotions. A multidisciplinary review on healthier eating strategies at restaurants found that the restaurants were responding to the increased customer demand for healthier offerings, but the response varied according to restaurant type and who initiated the change [31]. Although the most common strategy was the increase in healthy offerings and the promotion of such offerings, revenue concerns, and a lack of interest were the primary barriers among independently owned restaurants. A systematic review on international salt reduction policy in restaurants demonstrated the current common strategies, including menu labeling, target setting, and reformulation of dishes, education campaigns, chef training, toolkit delivery, table salt removal, media campaign, and government assistance such as free nutrition analysis and toolkit distribution [20]. However, the evaluations of these policies were limited, and the results were inconsistent. The present study integrated the activities on dimensions of consumer, chef, service staff, and environment, found the significant effects on promoting consumers’ awareness and behaviors, as well as the further explorations for chefs and waiters/waitresses to improving their knowledge, attitude, and practice to provide fewer sodium choices.

### 4.3. Strengths and Limitations of this Study

In an RCT with restaurants as study subjects, 192 restaurants, from 12 counties of 6 provinces were recruited for the study. In total, 96 restaurants in the intervention group carried out a series of comprehensive activities, including not only building restaurant environments that make it easier for the customers to choose lower salt options but also training for chefs and waiters/waitresses so as to improve the ability of salt-reduction service in the restaurants.

For the primary outcome, we collected the whole portion of the five best-selling dishes of each restaurant and tested their sodium content using the laboratory flame atomic absorption spectrometry method. We ensured an accurate assessment of the sodium content of the dishes.

A specially designed mobile EDC was used to collect assessment data during the study. The EDC system sets rules of logic jump for associated questions and abnormal value recognition so as to ensure the data validation and the detection of keying errors. All modifications were clearly recorded in the EDC system [23].

There were also several limitations. First, the comprehensive interventions in restaurants were unavoidably interrupted by COVID-19. All the restaurants were temporarily closed due to the pandemic of COVID-19 in early 2020, and the interruption of intervention may have influenced the outcomes. Furthermore, the losses during follow-up from the pandemic also limited the statistical power of the analysis. Second, although pre- and post-intervention outcomes were assessed, there was a lack of assessment at a different stage of the trial. Finally, the proportion of less-sodium orders, which was one of the secondary outcomes, was based on the interviews with the owners/managers who directly manage the restaurant. Although it was not based on direct statistics of restaurant dish sales, restaurant management should be able to be trusted with its assessment of the order of magnitude of less-sodium orders.

### 4.4. Public Health Implications

With the rapid urbanization and lifestyle changes in China, restaurants became the second major dining location after home cooking in China. As restaurant dishes have a high sodium content, it is important to develop a comprehensive reduction package for the sodium content of restaurant foods. The comprehensive sodium reduction package, which was established and used in this RCT, fills this gap and provides an effective tool for salt reduction in Chinese restaurants in the future. In China, some of the new public health policies, such as the guidelines on menu nutrition labeling for restaurant foods and the guidelines on the construction of healthy restaurants, also provide possible support for the promotion and application of the comprehensive sodium reduction package in restaurants. In the future, to refine the intervention package to increase its feasibility in the real world, practical initiatives should be established, such as providing standard communication words to the waitress for low sodium service, and standard training to chefs, as well as promoting the use of aromatic plants, such as herbs and spices, to increase food flavor and palatability to reduce salt intake.

## 5. Conclusions

Our study showed that the sodium content in restaurant meals is very high in China, and our education program led to an increase in consumers’ demand for lower sodium in their meals, whereas the overall reduction in the sodium levels of restaurant dishes and the use of salt and salty condiments by chefs were small and not statistically significant, compared with those in the control group. Further strengthening of the intervention package is needed to gradually reduce the large and unnecessary amount of salt and salty condiments added to restaurant food. There is great urgency for implementing an effective and sustainable salt reduction program in all restaurants, particularly due to the rapid increase in the consumption of restaurant food in China.

## Figures and Tables

**Table 1:** Baseline characteristics of restaurants.

Indicators	Control	Intervention	Total	*p*
**Basic information**				
Area (N, %) *				
North	32 (33.33)	32 (33.33)	64 (33.33)	1.0000
Central	32 (33.33)	32 (33.33)	64 (33.33)	
South	32 (33.33)	32 (33.33)	64 (33.33)	
Size (N, %)				
Large	25 (26.04)	23 (23.96)	48 (25)	0.8370
Medium	48 (50)	50 (52.08)	98 (51.04)	
Small	23 (23.96)	23 (23.96)	46 (23.96)	
Average cost (yuan/person) (N, %)				
<50	52 (54.17)	59 (61.46)	111 (57.81)	0.3063
≥50	44 (45.83)	37 (38.54)	81 (42.19)	
Monthly turnover (yuan/month) (N, %)				
<300,000	65 (67.71)	58 (60.42)	123 (64.06)	0.2924
≥300,000	31 (32.29)	38 (39.58)	69 (35.94)	
**Restaurant environment and service**				
Availability of nutritional materials (N, %)				
	39 (40.63)	33 (34.38)	72 (37.5)	0.3711
Less salt choice on menu (N, %)				
	14 (14.58)	17 (17.71)	31 (16.15)	0.5563
**Total**	96 (100)	96 (100)	192 (100)	

* Note: north (Heilongjiang and Qinghai), central (Hebei and Hunan), south (Jiangxi and Sichuan).

**Table 2:** Sodium content of restaurant dishes by laboratory test (mg/100 g).

Baseline (Mean (SD))		Change at 12 Months from Baseline (Mean (95% CI)) ^†^		Difference (95% CI) in Change between Intervention and Control, *p* Value		Adjusted Difference (95% CI) in Change between Intervention and Control, *p* Value ^‡^	
Control	Intervention	Control	Intervention				
**Including restaurants based on intention to treat analysis**							
540.9 (176.8)	551.9 (149.0)	−40.12 (−75.80–−4.45) *	−77.16 (−110.18–−44.14) *	−37.04 (−85.65–11.57)	0.13	−43.63 (−92.94–5.66)	0.08
**Including restaurants with complete laboratory test both at baseline and at the follow-up (*n* = 146)**							
538.4 (181.1)	545.7 (144.3)	−38.90 (−75.74–−2.05) *	−74.19 (−107.66–−40.72) *	−35.29 (−85.06–14.48)	0.16	−38.18 (−88.71–12.34)	0.13
**Including all restaurants at baseline using MI (*n* = 192)**							
540.9 (176.8)	551.9 (149.0)	−36.04 (−58.53–−17.45) *	−78.90 (−93.56–−67.03) *	−39.43 (−79.75–13.90)	0.11	−41.97 (−82.12–14.31)	0.09

SD = standard deviation; MI = multiple imputation. ^†^ Means and 95% confidence intervals apply to differences within each group between baseline and 12-month follow-up. ^‡^ Adjusted for restaurant size, area, cost, and turnover. * *p* < 0.05.

**Table 3:** Subgroup analysis of sodium content by laboratory test (mg/100 g).

	Baseline (Mean (SD))		Change at 12 Months from Baseline (Mean (95% CI)) ^†^		Difference (95% CI) in Change between Intervention and Control, *p* Value		Adjusted Difference (95% CI) in Change between Intervention and Control, *p* Value ^‡^	
	Control	Intervention	Control	Intervention				
**Size**								
Large	514.7 (176.3)	522.2 (125.0)	−38.15 (−103.49–27.17)	−101.09 (−169.65–−32.52) *	−62.93 (−157.64–31.77)	0.19	−69.18 (−164.74–26.37)	0.15
Medium	551.2 (189.7)	552.8 (163.4)	−35.70 (−86.16–14.75)	−77.51 (−124.60–−30.42) *	−41.80 (−110.83–27.21)	0.23	−40.45 (−110.00–29.08)	0.25
Small	548.2 (152.4)	579.6 (138.1)	−52.08 (−138.01 to 33.84)	−56.45 (−122.40 to 9.49)	−4.36 (−112.69 to 103.95)	0.93	−17.98 (−128.32 to 92.35)	0.74
**Area**								
North	483.8 (121.3)	522.0 (127.7)	−8.69 (−69.30–51.92)	−36.75 (−93.62–20.11)	−28.06 (−111.18–55.05)	0.50	−34.46 (−118.67–49.73)	0.41
Central	541.9 (159.2)	552.4 (179.9)	−55.97 (−115.55–3.61)	−93.84 (−150.71–−36.97) *	−37.87 (−120.25–44.48)	0.36	−39.77 (−122.93–43.38)	0.34
South	597.1 (221.7)	581.3 (132.6)	−52.42 (−118.00–13.15)	−101.42 (−159.14–−43.70) *	−49.00 (−136.36–38.35)	0.26	−56.30 (−144.47–31.86)	0.20

SD = standard deviation. ^†^ Means and 95% confidence intervals apply to differences within each group between baseline and 12-month follow-up. ^‡^ Adjusted for restaurant size, area, cost, and turnover. * *p* < 0.05.

**Table 4:** Use of salt and salty condiments in restaurant dishes (g/100 g), based on intention to treat analysis.

	Baseline (Mean (SD))		Change at 12 Months from Baseline (Mean (95% CI)) ^†^		Difference (95% CI) in Change between Intervention and Control, *p* Value		Adjusted Difference (95% CI) in Change between Intervention and Control, *p* Value ^‡^	
	Control	Intervention	Control	Intervention				
**Salt**	0.81 (0.05)	0.78 (0.05)	−0.04 (−0.16–0.07)	−0.13 (−0.24–−0.03) *	−0.09 (−0.25–0.07)	0.27	−0.09 (−0.25–0.07)	0.26
**Monosodium** **glutamate**	0.60 (0.04)	0.58 (0.04)	−0.15 (−0.22–−0.07)	−0.20 (−0.27–−0.13) *	−0.05 (−0.16–0.05)	0.31	−0.06 (−0.16–0.05)	0.28
**Soy sauce**	0.75 (0.05)	0.76 (0.05)	−0.07 (−0.16–0.02)	−0.15 (−0.23–−0.06) *	−0.08 (−0.20–0.05)	0.23	−0.08 (−0.21–0.05)	0.23
**Other condiments**	4.39 (0.21)	4.84 (0.21)	−0.68 (−1.02–−0.33) *	−0.86 (−1.18–−0.55) *	−0.19 (−0.66–0.28)	0.43	−0.22 (−0.69–0.25)	0.36

Abbreviation: SD, standard deviation. ^†^ Means and 95% confidence intervals apply to differences within each group between baseline and 12-month follow-up. ^‡^ Adjusted for restaurant size, area, cost, and turnover. * *p* < 0.05.

## Data Availability

The datasets generated and/or analyzed during the current study are not publicly available due to considerations of intellectual property. However, may be available from the corresponding author on reasonable request. HW affirm that the manuscript is an honest, accurate, and transparent account of the study being reported; that no important aspects of the study have been omitted; that any discrepancies from the study as planned (and, if relevant, registered) have been explained.

## Supplementary Materials

The following supporting information can be downloaded at: https://www.mdpi.com/article/10.3390/nu14245313/s1, Table S1: Framework of the intervention activities; Table S2: Baseline characteristics of follow-up and lost-restaurants; Figure S1: The implementation rate of restaurant interventions.
