# Supplementary material for: Sodium Reduction in Restaurant Food: A Randomized Controlled Trial in China
Source: Nutrients. 2022 Dec 14;14(24):5313. doi: 10.3390/nu14245313 (PMC9781955; doi:10.3390/nu14245313)
Supplement: Supplementary file 1 [file nutrients-14-05313-s001.zip › supplement tables.pdf]

Supplementary material

Table S1. Framework of the intervention activities

Activities	Description	Objectives	Frequency
1.Building supportive environment	1.1 Materials on sodium reductions such as videos, posters, brochures, leaflets and table displays were delivered regularly to restaurants and displayed at noticeable positions.<br>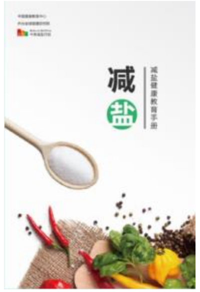<br>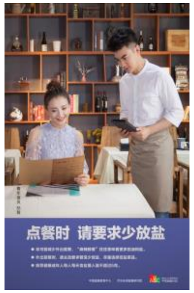<br>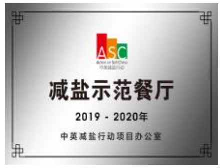<br>1.2 Give the intervention restaurants the brand medal of demonstration restaurants to build the salt reduction environment.	Promoting lower sodium choices.<br>Empowering cognition for consumers.	Throughout the whole trial period.
2. Activities on consumers	2.1 Materials in supportive environment<br>2.2 Lower-salt menu labelling for 10% dishes with lowest sodium content in each restaurant.<br>2.3 Salt reduction selection tips on menu/order tables	Convey the idea “less salt more health” to the consumers.<br>Guide consumers to make lower-sodium choices.<br>Promote consumers to request salt reduction services by providing multiple choice for salt used	Throughout the whole trial period.

	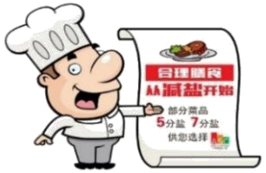	(ie. Normal salt, 70% slat or 50% salt).	
3. Enhance chefs' cooking skills to reduce sodium	3.1 Face-to-face training by nutritionist and culinary experts;<br>3.2 Series training materials to chefs, including manual and videos, comprising lessons on: 'sources of sodium in restaurant meals', 'why reducing sodium', 'practical cooking skills to reduce sodium'.<br>3.3 Monthly supervision by local investigators.<br>3.4 low- sodium dishes reformulation<br>3.5 Encourage chefs to reduce sodium by at least 10% in all dishes and more reductions according to customers' request.	Transfer knowledge and skills among chefs.<br>Enhance the knowledge and skills regularly.<br>Promote the practice of active salt reduction.<br>Provide more low-/lower-sodium dishes in restaurants.	3.1 Once per year<br>3.2 Throughout the whole trial period.<br>3.3 Once per month<br>3.4 Reformulate at least three low-sodium dishes (sodium≤100mg/100g) per restaurant during the trial period.<br>3.5 Throughout the whole trial period.
4. Encourage waiters/waitresses to proactively remind and introduce lower-sodium choices in ordering services	4.1 Face-to-face training by nutritionist and culinary experts;<br>4.2 Series training materials, including manual and videos on "salt sources in restaurant dishes", "why reduce salt", "building a reduced-salt environment in restaurants" and "service and communication skills.<br>4.3 Monthly supervision by local investigators.<br>4.4 Record and deliver accurately the customer's request for less salt.	Transferring knowledge and skills among waiters/waitresses.<br>Enhance the knowledge and skills regularly.<br>Provide active and effective salt reduction services in ordering.	4.1 Once per year.<br>4.2 Throughout the whole trial period.<br>4.3 Once per month.<br>4.4,4.5 Throughout the whole trial period.

	4.5 Encourage waiter/waitress to recommend the lower-sodium dishes, and remind customers choosing the less salt option.

Table S2. Baseline characteristics of follow-up and lost-restaurants.

Indicators	Follow-up	Lost	Total	*P*
**Basic information**				
Area [a] （N, %）				
North	50 (34.25)	14 (30.43)	64 (33.33)	
Central	51 (34.93)	13 (28.26)	64 (33.33)	0.3017
South	45 (30.82)	19 (41.3)	64 (33.33)	
Size (N, %）				
Large	40 (27.4)	8 (17.39)	48 (25)	
Medium	74 (50.68)	24 (52.17)	98 (51.04)	0.1183
Small	32 (21.92)	14 (30.43)	46 (23.96)	
Average cost (yuan/person) （N, %）				
<50	83 (56.85)	28 (60.87)	111 (57.81)	0.6311
≥50	63 (43.15)	18 (39.13)	81 (42.19)	
Monthly turnover (yuan/month) （N, %）				
<300000	91 (62.33)	32 (69.57)	123 (64.06)	0.3737
≥300000	55 (37.67)	14 (30.43)	69 (35.94)	

**Restaurant environment and service**				
Availability of nutritional materials（N, %）				
	58 (39.73)	14 (30.43)	72 (37.5)	0.4776
Less salt choice on menu（N, %）				
	22 (15.07)	9 (19.57)	31 (16.15)	0.2514
**Total**	146 (100)	46 (100)	192 (100)	

[a] North (Heilongjiang and Qinghai), Central (Hebei and Hunan), South (Jiangxi and Sichuan).
