# Supplementary figures and images for: Sodium Reduction in Restaurant Food: A Randomized Controlled Trial in China
Source: Nutrients. 2022 Dec 14;14(24):5313. doi: 10.3390/nu14245313 (PMC9781955; doi:10.3390/nu14245313)

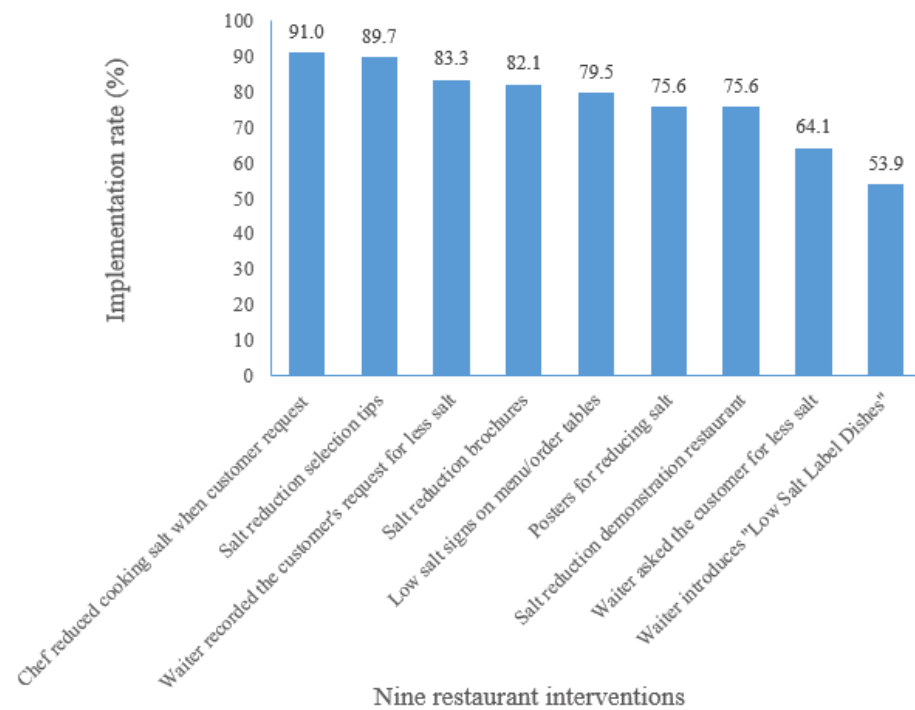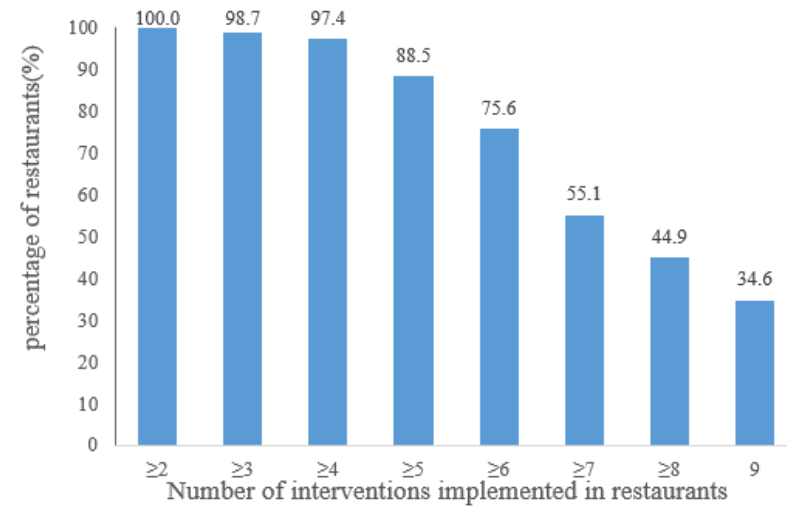

**FIGURE S1 The implementation rate of restaurant interventions**

Supplement: Supplementary file 1 [file nutrients-14-05313-s001.zip › supplement figure S1.pdf]
